# Therapeutic drug monitoring of liposomal amphotericin B in children. Are we there yet? A systematic review

**DOI:** 10.1093/jac/dkae003

**Published:** 2024-01-22

**Authors:** Tony Lai, Chin-Yen Yeo, Bradley Rockliff, Michael Stokes, Hannah Yejin Kim, Ben J Marais, Andrew J McLachlan, Jan-Willem C Alffenaar

**Affiliations:** Pharmacy Department, The Children’s Hospital at Westmead, Sydney, NSW, Australia; The University of Sydney Infectious Diseases Institute (Sydney ID), Sydney, NSW, Australia; Sydney Pharmacy School, Faculty of Medicine and Health, The University of Sydney, Sydney, NSW, Australia; Pharmacy Department, Concord Hospital, Sydney, Australia; Pharmacy Department, The Children’s Hospital at Westmead, Sydney, NSW, Australia; Pharmacy Department, The Children’s Hospital at Westmead, Sydney, NSW, Australia; The University of Sydney Infectious Diseases Institute (Sydney ID), Sydney, NSW, Australia; Sydney Pharmacy School, Faculty of Medicine and Health, The University of Sydney, Sydney, NSW, Australia; Pharmacy Department, Westmead Hospital, Sydney, Australia; The University of Sydney Infectious Diseases Institute (Sydney ID), Sydney, NSW, Australia; Sydney Pharmacy School, Faculty of Medicine and Health, The University of Sydney, Sydney, NSW, Australia; The University of Sydney Infectious Diseases Institute (Sydney ID), Sydney, NSW, Australia; Sydney Pharmacy School, Faculty of Medicine and Health, The University of Sydney, Sydney, NSW, Australia; Pharmacy Department, Westmead Hospital, Sydney, Australia

## Abstract

**Introduction:**

Therapeutic drug monitoring (TDM) is a tool that supports personalized dosing, but its role for liposomal amphotericin B (L-amb) is unclear. This systematic review assessed the evidence for L-amb TDM in children.

**Objectives:**

To evaluate the concentration–efficacy relationship, concentration–toxicity relationship and pharmacokinetic/pharmacodynamic (PK/PD) variability of L-amb in children.

**Methods:**

We systematically reviewed PubMed and Embase databases following PRISMA guidelines. Eligible studies included L-amb PK/PD studies in children aged 0–18 years. Review articles, case series of <five patients, editorials and animal studies were excluded. Quality assessment was performed using the Critical Appraisal of Clinical Pharmacokinetics tool. The concentration–efficacy and concentration–toxicity relationships and PK/PD variability were analysed.

**Results:**

In total, 4220 studies were screened; 6 were included, presenting data on 195 children. Invasive candidiasis and aspergillosis were the two most common infections treated with L-amb. Studies showed significant PK variability due to age (mean age ranged from 14 days to 17 years), body weight, non-linear PK and changes in the volume of distribution. Limited evidence supported a peak concentration/MIC (*C*_max_/MIC) of 25–50 for optimal efficacy and an AUC_24_ of >600 mg·h/L for nephrotoxicity. L-amb doses of 2.5–10 mg/kg/day were reported to achieve *C*_max_/MIC > 25 using an MIC of 1 mg/L.

**Conclusions:**

While significant PK variability was observed in children, evidence to support routine L-amb TDM was limited. Further studies on efficacy and toxicity benefits are required before routine TDM of L-amb can be recommended.

## Background

There have been substantial advances in treating children with leukaemia that have improved cancer survival; however, there is limited progress in treating invasive fungal disease (IFD).^1^ IFD is a common complication in children treated with leukaemia and has significant attributable mortality.^2^ Liposomal amphotericin B (L-amb) is a critical anti-infective to treat serious IFD. Preclinical studies have shown L-amb has concentration-dependent activity, with the pharmacokinetic/pharmacodynamic (PK/PD) index being the maximum amphotericin B (amB) concentration/MIC ratio (*C*_max_/MIC) of 25 to 50,^3,4^ Although L-amb has been used in clinical practice for decades, human studies have not confirmed the correlation between L-amb concentrations and efficacy or toxicity (such as hypokalaemia and nephrotoxicity).^3^ Thus, the evidence to support the implementation of therapeutic drug monitoring (TDM) to guide dosing is limited^3^ and, therefore, not routinely recommended.^5–7^ As the body of evidence on antifungal TDM is increasingly adopted as the standard of care for specific patient populations, it is worthwhile reassessing TDM for L-amb.^8,9^ This is especially the case in children where PK variability and optimal dosing to ensure efficacy and toxicity are poorly defined.^10–13^ This is further supported as IFD still has unacceptably high treatment failure rates and significant toxicity despite adequate drug selection.^3,14,15^ Moreover, evidence for L-amb use is based on randomized controlled trials (RCTs) on adults, and it is clear that the PK of L-amb is different in children.^15–19^ Therefore, this systematic review aimed to evaluate if the accumulated evidence of the L-amb concentration–efficacy relationship, concentration–toxicity relationship and PK variability in children supports the implementation of TDM to optimize patient outcomes from L-amb in this vulnerable group of patients.

## Methods

### Search strategy and selection

This systematic review was conducted according to PRISMA guidelines.^20^ All observational (both retrospective and prospective) and interventional studies that evaluated the PK/PD of L-amb in children and adolescents aged 0–18 years were eligible for inclusion in our study. Studies of L-amb not administered IV, and non-liposomal formulations were excluded. In addition, review articles, commentaries, editorials, animal studies and case series of fewer than five patients were excluded.

We searched PubMed and Embase for relevant studies from 1 January 1984 to 31 July 2023 using a combination of the following MeSH terms: (‘Amphotericin B)’ AND ‘(Drug monitoring’ OR ‘Pharmacokinetics’ OR ‘Pharmacodynamics’ OR ‘drug concentration’) AND (‘child’ OR ‘adolescent’). A human filter was applied, and only studies in the English language were included. See Tables S1 and S2 (available as Supplementary data at *JAC* Online) for the detailed search strategy.

Retrieved articles were uploaded to COVIDENCE (https://www.covidence.org/) and duplicates were removed. Titles and abstracts were screened for eligibility, followed by a full-text review of selected studies by independent reviewers (T.L., C.Y.Y., M.S. and B.R.). Discrepancies between reviewers were settled by discussion and a third reviewer (T.L. or J.W.C.A.). The reason for the study exclusion was documented during the full-text review. The reference lists of all included full-text articles were searched to identify any other eligible studies not captured in the search strategy.

### Quality assessment

Two independent reviewers (T..L. and C.Y.Y.) used a validated Critical Appraisal of Clinical Pharmacokinetic tool (CACPK) to evaluate the quality of studies.^21,22^ This tool consists of 21 questions, with each item being either 1 for ‘yes’ or 0 for ‘I do not know’, ‘no’, or ‘not applicable’. High quality is a score of 13 or higher; scores of 12–13 and less than 12 were considered fair to moderate and poor, respectively.^21,22^ The quality assessment tool is found in Table S3. Any discrepancies or lack of consensus regarding the quality assessment by the first and second reviewers were mediated by a third independent reviewer (J.W.C.A.).

### Data extraction

Data extracted from eligible studies include year of publication, study type, age, number of patients, L-amb assay used (total or free drug levels), indication (treatment or prophylaxis), dose, number of sampling timepoints and PK parameters AUC_24_, *C*_max_, *t*_½_, *V*_d_ and CL. Lastly, we analysed any descriptions of concentration–efficacy relationships, concentration–toxicity relationships, PK variability, non-linear PK and recommendations of TDM.

## Results

### Included studies

**Figure 1.**
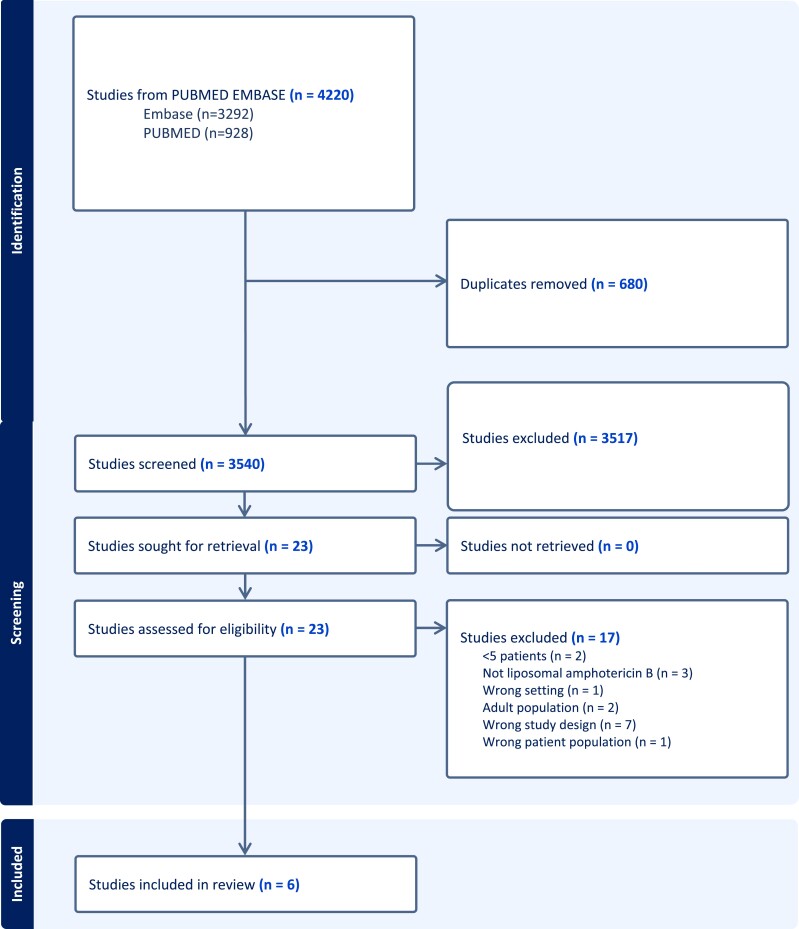
PRISMA flowchart of studies included in the review reporting PK/PD data for liposomal amphotericin B use in children. This figure appears in colour in the online version of *JAC* and in black and white in the print version of *JAC*.

The literature search identified 4220 publications (3292 Embase and 928 PubMed), and of these, 680 duplicates were removed, leaving 3450 articles for abstract screening. Twenty-three articles were selected for full-text screening, of which 17 were excluded, resulting in six articles for extraction (Figure 1). All six studies were assessed for quality using CACPK. Half of the studies (3/6) were evaluated as high quality, with two being moderate/fair and one being poor quality (Table 1). Kotwani *et al.*^26^ rated poor quality due to the lack of descriptions of the objectives or methods of the study, and there were no justifications for the study design.

**Table 1.** Characteristics and PK/PD findings from studies assessing L-amb use in children

Study (year)	Type	CACPK quality grading	Number of paediatric participants	Number of plasma samples	Age (years) (range)	L-amb assay used	Indication	Dose (mg/kg/day)	*C*_max_ (mg/L)	*C*_min_ (mg/L)	AUC_24_ (mg·h/L)	*t*_½_ (h), mean (SD)	CL (mL/hr/kg)	V (L/kg)
Ohata *et al.* (2015)^23^	NS	Moderate/fair (13/21)	39	159 (2–7 timepoints)	8.4 (8 months to 15 years)	HPLC (total)	Treatment	1 2.5 5	— 17 (7.6) NS	— — —	— — —	— — —	— — —	— — NS
Mehta *et al.* (2006)^24^	Prospective single centre	Moderate/fair (13/21)	14	NS (13 timepoints)	3^a^ (4 months to 9 years)	Bioassay (free)	Prophylaxis	10	2.71 (0.47)	0.23 (0.13)	156.21 (69.73)	43.42 (14.11)	0.07 (0.03)	4.19 (1.14)
Hong *et al.* (2005)^25^	Prospective single centre	High (17/21)	39	637 (13 timepoints)	7.1 (2 months to 17 years)	HPLC (total)	Treatment	1 3 5	— — —	— — —	— — —	— — —	— — —	— — —
Kotwani *et al.* (2002)^26^	NS	Poor (3/21)	28 (17 neonates)	22 (2 timepoints)	NS (14 days to 12 years)	HPLC (total)	Treatment	1 (neonate) 1 (>30 days age)	0.54 (0.17) 0.63 (0.2)	0.22 (0.04) 0.24 (0.12)	— —	— —	— —	— —
Lestner *et al.* (2016)^27^	Prospective multicentre	High (20/21)	35	— (7–12 timepoints)	8.7 (1–17 years)	HPLC (total)	Treatment	2.5 5 7.5 10	— — — —	— — — —	— — — —	— — — —	— — — —	— — — —
Seibel *et al.* 2017^b28^	Prospective multicentre	High (20/21)	40	— (9 timepoints)	7.8 (NS)	HPLC (total)	Treatment	2.5 5 7.5 10	49.5 (31.9) 64.1 (45.8) 57.4 (24.0) 83.1 (48.9)	— — — —	301 (180) 767 (1115) 395 (216) 786 (689)	14.3 (7.5) 17.9 (4.8) 21.3 (8.8) 27.4 (23.5)	10 (6) 13 (12) 17 (7) 17 (11)	0.2 (0.16) 0.31 (0.2) 0.53 (0.35) 0.63 (0.51)

—, not stated; pt, patient; NS not stated/studied.

^a^Age, mean or median (range).

^b^Seibel *et al.* (2017)^28^ PK parameters at last day of treatment.

### Details of selected studies

A total of 195 children and adolescents who had received L-amb from six studies were included in the review.^23–28^ The sample sizes of the included studies ranged from 14 to 40 participants, and the mean age of participants in the studies ranged from 14 days to 17 years. L-amb was indicated for treatment in 92.8% (181/195) of the patients rather than prophylaxis of IFD. Candidiasis (53%, 9/17) and aspergillosis (29%, 5/17) were the two most common infections, followed by single cases of infections due to *Cryptococcus neoformans*, *Lomentospora prolificans* and *Rhodotorula rubra*.

**Figure 2.**
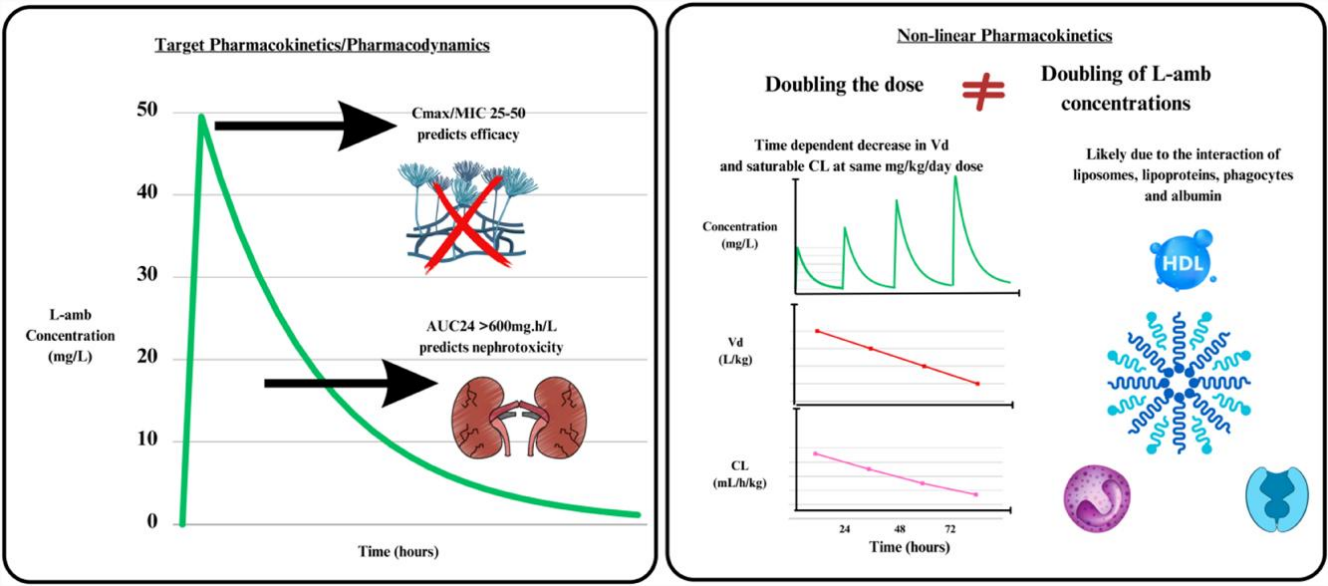
Summary of key findings related to pharmacokinetic/pharmacodynamic indices and non-linear PK of liposomal amphotericin B, likely due to saturable interactions as described, with variability related to age and nutritional status. This figure appears in colour in the online version of *JAC* and in black and white in the print version of *JAC*.

The number of PK samples per patient ranged from 2 to 13, and the richest PK-sampled study was by Hong *et al.*,^25^ with 637 samples in 39 participants. Most studies used HPLC with UV spectroscopy with total (protein-bound + unbound) amB concentrations (5/6).^23,25–28^ In contrast, one used a bioassay with only unbound amB concentrations.^24^ Only two studies reported PK parameters (e.g. AUC, *C*_max_, *C*_min_, CL, *V*_d_ or *t*_½_).^24,28^ The description of the concentration–efficacy relationship, concentration–toxicity relationships, PK variability and recommendation of TDM of the included studies are summarized in Table 2 and Figure 2.

**Table 2.** Summary of factors supporting routine L-amb TDM in children

Study (year)	Pharmacokinetic variability	Concentration–efficacy relationship	Concentration–toxicity relationship	Routine TDM recommendation
Ohata *et al.* (2015)^23^	Significant variability of *C*_min_ at doses of 1, 2.5 and 5 mg/kg/day due to saturable clearance	NS	No relationship between *C*_min_ and hypokalaemia	NS
Mehta *et al.* (2006)^24^	Non-linear elimination	NS	NS	NS
Hong *et al.* (2005)^25^	Body weight <20 kg had lower *C*_max_	*C*_max_/MIC > 50 against *Candida* and *Aspergillus*	NS	‘Further work is needed to clarify the possible impact of this observation on the TDM of L-Amb in children.’
Kotwani *et al.* (2002)^26^	Significant variability of *t*_½_, *V*_d_ and CL in neonates	NS	NS	NS
Lestner *et al.* (2016)^27^	Non-linear PK >7.5 mg/kg Time-dependent reduction of *V*_d_	NS	AUC_24 _> 600 mg·h/L and nephrotoxicity	TDM likely to be of value to prevent nephrotoxicity
Seibel *et al.* (2017)^28^	Significant interpatient variability *V*_d_ and CL Non-linear PK at doses >2.5 mg/kg	No relationship found	Higher doses (7.5–10 mg/kg/day) increased creatinine, hypokalaemia and infusion-related reactions^a^	NS

NS, not stated/studied.

^a^Seibel *et al.* (2017)^28^ found a dose–toxicity relationship. The AUC_24_ and *C*_max_ at doses of 7.5–10 mg/kg/day were higher at these doses compared with doses of 2.5–5 mg/kg/day.

### AmB concentration–efficacy relationship

Only two of the six studies investigated the data on the concentration–efficacy relationship of L-amb in children: Hong *et al.*^25^ and Seibel *et al.*^28^

The Hong *et al.*^25^ study was a prospective observational PK study of 39 paediatric patients with cancer. They investigated the amB *C*_max_/MIC and its relationship with the clinical efficacy of nine patients with proven fungal infections administered L-amb. The mycological diagnosis of these infections were predominately yeast infections [(4/9) consisting of *Candida albicans* (3/9), *Pichia kudriavzevii* (previously known as *Candida krusei*) (1/9)] and *Aspergillus fumigatus* infections (2/9). One patient had a proven polyfungal infection with *C. albicans*, *A. fumigatus* and *Aspergillus niger.* Another patient had a proven CNS infection with *L. prolificans* (previously known as *Scedosporium prolificans*) cultured in the CSF. Most patients (*n* = 30/39) were excluded from the concentration–efficacy analysis because they had possible or probably fungal infections or received empirical treatments lacking MIC data. Eight of the nine patients achieved clinical response [(complete *n* = 4) or partial (*n* = 4)] except the one patient with *L. prolificans* isolated in the CSF who failed to respond. All nine patients received doses of 3–5 mg/kg/day regardless of whether it was a yeast or mould infection. The authors of this study found that *C*_max_/MIC > 50 assessed at steady state (Day 7) was statistically higher in the patients with a complete response compared with patients with a partial response (*P* = 0.021). Lastly, these researchers found no correlation between L-amb AUC/MIC assessed at steady state and response to treatment (*P* = 0.285).

Seibel *et al.*^28^ did not identify a clear relationship between concentration–efficacy in a prospective multicentre PK study investigating escalating doses of L-amb (2.5, 5, 7.5 and 10 mg/kg/day) in 40 immunocompromised children and adolescents. In this study, 82.5% (33/40) patients were treated with L-amb empirically, and 17.5% (7/40) received directed treatment. Five patients (5/7) had proven fungal disease, but no clear mycological description of fungal species or MIC was described. The remaining two patients (2/7) with proven fungal infection had *C. albicans* and *Candida parapsilosis* with no MICs reported. Overall, 25/40 of patients who had received empirical or directed treatment with L-amb had a successful outcome, defined as ‘resolution/improvement of fever and clinical signs and symptoms’ for empirical treatment or based on ‘clinical, radiological and microbiological criteria’ for directed treatment. The remaining 8/40 patients experienced treatment failure, defined as ‘death, breakthrough fungal infection, or withdrawal due to an adverse event.’ The authors found no treatment failures in the patients who received 10 mg/kg/day. However, no trend of a dose–response relationship in survival or efficacy could be demonstrated. Lastly, Seibel *et al.*^28^ applied preclinical murine PK/PD targets of amB *C*_max_/MIC = 25–50 to all four doses studied (2.5, 5, 7.5 and 10 mg/kg/day). Using a *C. albicans* MIC of 0.25 mg/L, these researchers found that all four L-amb doses achieved the PK/PD target of 25–50 but did not investigate this relationship further.

### AmB concentration–toxicity relationship

Three out of the six studies included studies that investigated a concentration–toxicity relationship: Ohata *et al.*,^23^ Seibel *et al.*^28^ and Lestner *et al.*^27^

Ohata *et al.*^23^ performed a retrospective PK study of 39 patients. They investigated the relationship between amB *C*_min_ and toxicity in 18/39 patients. This study defined toxicity as hypokalaemia, a decrease in potassium of ≤80% from baseline pretreated with L-amb. Seven of the 18 (38%) reported hypokalaemia due to L-amb, with doses ranging from 1, 2.5 and 5 mg/kg/day. They found no relationship between *C*_min_ and hypokalaemia.

Seibel *et al.*^28^ conducted a multicentre prospective sequential dose-escalation PK study of 40 children given a range of L-amb doses (2.5, 5, 7.5 and 10 mg/kg/day). This study suggests a concentration–toxicity relationship (e.g. increased creatinine, hypokalaemia and infusion-related vomiting). Doses of >5 mg/kg/day L-amb produced higher PK exposures (AUC_24_ and *C*_max_) than doses of 2.5 mg/kg/day. They were associated with a 1.5–1.8× increase in creatinine (*P* < 0.05). They also found that higher doses of 10 mg/kg/day, and thus higher PK exposures (AUC_24_ and *C*_max_), showed a trend toward greater hypokalaemia and vomiting.

Lestner *et al.*^27^ investigated in a multicentre prospective PK study (*n* = 35) the relationship between PK exposure [*C*_max_, *C*_min_, L-amb dose (both absolute and relative to weight), AUC_24_] and toxicity. Toxicity was defined as nephrotoxicity (serum creatinine of ≥0.5 mg/dL or doubling of baseline value), hypokalaemia (potassium of ≤3.0 mmol/L or ≥50% from baseline), anaemia (haemoglobin of ≤8.0 g/dL) and hepatoxicity (rise in bilirubin by ≥1.5 mg/dL or AST or ALT ≥3 times above baseline). Nephrotoxicity and hypokalaemia were common in their study (46% and 23%, respectively) at a range of doses from 2.5, 5, 7.5 and 10 mg/kg/day. The authors of this study found a statistically significant relationship between AUC_24_ and nephrotoxicity (OR, 2.37; 95% CI, 1.84 to 3.22; *P* = 0.004). The Bayesian estimated linear regression suggests an AUC of >600 mg·h/L threshold for nephrotoxicity (increased serum creatinine of ≥0.5 mg/dL).

### PK variability

Hong *et al.*^25^ described body weight as a statistically significant contributor to PK variability of L-amb in children. The authors did a series of PK simulations of weight ranges from 10 to 70 kg at 1 to 12.5 mg/kg doses of L-amb and the differences in amB *C*_max_ at steady state. A key finding was that younger patients weighing less than 20 kg achieved statistically lower amB *C*_max_ at steady state compared with those having body weights of 20 kg and above. Moreover, it led to suboptimal PK exposure and higher mg/kg dosing requirements supported by Monte Carlo simulations. These simulation studies showed that ≥85% of children achieved amB *C*_max_/MIC > 50 at doses of 4–7.5 mg/kg/dose for *Candida* (MIC 0.5 mg/L) and *Aspergillus* (MIC 1.0 mg/L) only if the patients weighed more than 30 kg; lighter patients required higher L-amb doses of >7.5–12.5 mg/kg/dose to achieve *C*_max_/MIC > 50.

The significant impact of body weight on amB PK and L-amb dosing was corroborated by Lester *et al.*^27^ Children in this study had variable amB *C*_max_ and AUC_24_ when given the same doses. The authors discovered non-linear relationships between *C*_max_ versus mg/kg/dose and AUC_24_ versus mg/kg/dose.

Lester *et al.*^27^ described L-amb displays as ‘non-classical’ non-linear PK different from typically described with Michaelis–Menten kinetics. They found that L-amb in children exhibits a time-dependent reduction in the *V*_d_ with time. Contrasting intra- and interpatient PK variability was seen in their study, where concentration–time curves of one child showed stable *V*_d_ over 5 days. However, another child displayed a reduction in *V*_d_ four times (e.g. from 8L on Day 1 to 2L on Day 6) throughout a treatment course despite a consistent dose of L-amb 10 mg/kg/day. The reduction in *V*_d_ with time translated to increased PK exposures (e.g. AUC_24_ and *C*_max_) despite being on the same mg/kg L-amb dose throughout treatment. There were similar findings by Seibel *et al.*,^28^ who found a high degree of interpatient variability of PK parameters (AUC, *C*_max_, *t*_½_, *V*_d_ and CL). The PK parameters of the first day of L-amb treatment were compared with the last day (median 10 days, ranging from 3 to 42 days). The *V*_d_ had a 2–3-fold reduction over time at L-amb doses of 2.5, 5 and 7.5 mg/kg/day but not at 10 mg/kg/day. Like the findings from the study by Lester *et al.*,^27^ this increased L-amb PK exposures (*C*_max_ and AUC_24_) in children, even though the same mg/kg dose was administered throughout treatment. The authors of this study hypothesize that this variability is due to HDL saturation and phagocyte uptake.

Ohata *et al.*^23^ reported significant variable PK exposures at L-amb doses of 1, 2.5 and 5 mg/kg/day in children. They found a ≥10-fold increase in amB *C*_min_ after 5 days in up to 28% of children when given the same dose throughout treatment. They hypothesized that this was due to a saturable clearance pathway of the liposomes specific to the L-amb formulation. These researchers attribute the PK variability to different degrees of saturable uptake of amB by phagocytic activity in the reticuloendothelial system (RES). Despite this cohort of children with ≥10-fold increase in *C*_min_, it was not associated with hypokalaemia. This study did not investigate nephrotoxicity associations with *C*_min_.

The findings of Ohata *et al.*^23^ regarding saturable clearance were consistent with those of the study by Seibel *et al.*,^28^ which showed that L-amb clearance decreased with the duration of treatment. This finding was seen after multiple doses when comparing the first day with the last day of L-amb treatment at doses of 2.5, 5, 7.5 and 10 mg/kg/day. The clearance was reduced by 3–4 times across all four studied doses and contributed to increased *C*_max_ and AUC_24_ in children on the same dose consistently from the first and last day of treatment.

In addition to the time-dependent saturable clearance, Seibel *et al.*^28^ found a dose-dependent saturable clearance up to a dose of 7.5 mg/kg/day. L-amb clearance increased gradually at doses of 2.5, 5 and 7.5 mg/kg/day but not at 10 mg/kg/day.

Seibel *et al.*^28^ continued to show non-linear PK where an increased L-amb dose did not show a proportional increase in PK exposure (e.g. AUC_24_). After one dose, the children on 2.5 mg/kg/dose had a mean amB AUC_24_ of 54.7 mg/mL·h. However, the children on 5 mg/kg/dose had a mean AUC_24_ of 351 mg/mL·h. A dose-proportional increase in exposure would be expected in drugs with linear PK. The authors discuss that the increase in AUC_24_ is consistent with saturable non-linear clearance.

## Discussion

To our knowledge, this is the first systematic review evaluating the evidence to support L-amb TDM in children based on the concentration–efficacy relationship, concentration–toxicity relationship and factors contributing to the PK variability. The key findings of this study are that there is significant PK variability of L-amb, which justifies TDM in children. Based on limited evidence, an amB *C*_max_/MIC of 25–50 as a predictor for efficacy and AUC > 600 mg·h/L as a predictor for nephrotoxicity is proposed for L-amb. However, adequately powered prospective studies are required to validate these targets.

Firstly, there are several issues to consider when investigating an amB *C*_max_/MIC of 25–50 as a PK/PD target for L-amb efficacy in children. The evidence to support this target is limited to one study of a small subset of children (*n* = 9) that was not powered nor appropriately designed to define the PK/PD of L-amb. Hong *et al.*^25^ compared two groups of patients [partial response (*n* = 4) versus complete response (*n* = 6)] to find a *C*_max_/MIC > 50 for efficacy. They did not include treatment failures because only one patient failed L-amb treatment.

Furthermore, Hong *et al.*^25^ used unconventional definitions of treatment success versus treatment failure. They compared partial versus complete responses as categorical outcome groups, which is inconsistent with the comparison groups in modern antifungal RCTs and consensus group definitions.^15,29,30^ It would be more consistent to define treatment success as ‘a partial or complete response’ and define treatment failure as ‘stable or progression or death’. If these current and valid definitions of IFD treatment success were used, the PK/PD index *C*_max_/MIC could be lower than 50 in this study.

A *C*_max_/MIC of 25–50 is consistent with preclinical murine models for *C. albicans* given L-amb.^4^ One of the included studies by Seibel *et al.*^28^ discusses the use of the preclinical PK/PD index and reports that all the doses used in their study (e.g. 2.5, 5, 7.5 and 10 mg/kg/dose) achieved a *C*_max_/MIC of 25–50 using a *C. albicans* MIC of 0.25 mg/L. A *C*_max_ of 6.25–12.5 mg/L was required to achieve this target, and all the doses studied had a mean *C*_max_ above this range (49.5, 64.1, 57.4 and 83.1 mg/L, respectively)

This raises the controversial issue of which MIC to use as an L-amb PK/PD index denominator. Hong *et al.*^25^ reported Monte Carlo simulations using a *Candida* spp. MIC of 0.5 mg/L and *Aspergillus* spp. MIC of 1.0 mg/mL, but Seibel *et al.*^28^ used a lower *C. albicans* MIC of 0.25 mg/L. A 2–4-fold difference in the denominator results in a significant difference in PK/PD target attainment, thus leading to substantial differences in dosing requirements. CLSI and EUCAST are the primary reference mycological lab methodologies, but only EUCAST has developed breakpoints.^31^ The rationale for EUCAST clinical breakpoints for amB recommends an MIC breakpoint of 1 mg/L for *A. fumigatus*, *A. niger*, *C. albicans*, *Candida dubliniensis*, *Candida glabrata*, *C. parapsilosis*, *Candida tropicalis*, *P. kudriavzevii (previously known as C. krusei)* and *C. neoformans* and should be considered as the denominator for future studies in this research space.^32^ This simplifies TDM because a *C*_max_ of 25–50 mg/L would be required to meet target PK/PD using EUCAST MIC breakpoints.

Another considerable issue is the interchangeability of the PK/PD index for conventional and L-amb formulations. Preclinical murine candidiasis studies describe an amB *C*_max_/MIC ratio of 5–10, which results in fungicidal activity for the conventional deoxycholate amphotericin B (DamB). Andes *et al.*^33^ highlighted the 5-times potency of L-amb to DamB in preclinical murine studies and is consistent with the PK/PD targets described in the included clinical studies of this systematic review. The differences between the PK/PD of L-amb and DamB are underemphasized, such that a lower DamB *C*_max_/MIC ratio of 5–10 is improperly applied to studies using L-amb. One L-amb paediatric TDM study by Tortora *et al.*,^34^ which was excluded due to the paediatric patient numbers (<5), and another adult multicentre study currently underway by Roberts *et al.*,^35^ use a lower *C*_max_/MIC target of >4.5 for L-amb.

When investigating an AUC_24_ as a predictor for nephrotoxicity for L-amb in children, there are issues to consider. The nephrotoxicity mechanism has been described as a combination of factors (e.g. oxidative stress, disturbance of potassium homeostasis, vasoconstriction of renal vasculature).^36,37^ The mechanism is thought to be related to higher doses and longer duration, which makes it plausible to be driven by AUC_24_ exposure.^38,39^ The assessment of the concentration–toxicity relationship was limited to one study, by Lestner *et al.*,^27^ where they found a statistically significant relationship between AUC_24_ and nephrotoxicity (OR, 2.37; 95% CI, 1.84 to 3.22; *P* = 0.004). Unfortunately, the study was limited by not measuring potential confounders of renal impairment (e.g. disease severity and concomitant nephrotoxic drugs). This study was conducted in immunocompromised children where nephrotoxic drugs are commonly given (e.g. aminoglycosides, vancomycin, aciclovir and chemotherapy).^40^ The authors of this study used different definitions of acute kidney injury (AKI) to the Kidney Disease: Improving Global Outcomes (KDIGO) criteria.^41^ The KDIGO criteria define AKI as either an increase in serum creatinine (SCr) by 50% within 7 days or an increase in SCr by 0.3 mg/dL (26.5 mmol/L) within 2 days or oliguria. Lestner *et al.*^27^ used a higher threshold level of definitions (e.g. increase in SCr of 100% or SCr by 0.5 mg/dL with no specific days of occurrence), and AKI commonly occurred in 46% (16/35) of children in this study. If KDIGO criteria were used, a higher percentage would be expected. If KDIGO definitions were applied, an AUC >500 mg·h/L, rather than >600 mg·h/L, should be studied further as the threshold for AKI.

Our systemic review found several factors contributing to L-amb’s PK variability that would support TDM in children. These include lower body weight (and by extrapolation, age), non-linear PK due to a reduced volume of distribution and saturable clearance with time of therapy. The studies analysed in this systematic review described the time-dependent non-linear PK that led to increased exposures (e.g. *C*_max_ and AUC_24_). Unfortunately, these studies were not powered or designed to define a saturation timepoint where the changes in PK led to an increased risk of toxicity (e.g. AKI). A Kaplan–Meier curve from a large RCT of L-amb in children (*n* = 204) has described 1 and 3 mg/kg/day causing similar AKI rates until Day 14. These data may provide a hypothesis for the time-dependent PK saturation point that translates to nephrotoxicity.^39^ Lestner *et al.*^27^ theorize that the PK variability is driven by variable saturation points of lipoproteins and/or phagocyte uptake, which were not characterized due to the small patient numbers. L-amb is a highly protein-bound and complex process of albumin, HDL and WBC reservoirs, which has led researchers to recommend that total L-amb levels be measured rather than free (unbound) levels.^42,43^ However, this recommendation was received with controversy.^44^ Most studies (5/6) in this systemic review used total levels to describe the PK/PD relationships. Future studies must consider the effect of fluctuating HDL concentrations, albumin and WBC counts on L-amb PK in children using total levels.

The studies in this review highlight that more work needs to be done to establish the role of L-amb TDM. The accumulated evidence from the analysed studies suggests L-amb is a candidate drug for TDM (e.g. marked variability due to non-linear PK, concentration–efficacy relationship and concentration–toxicity relationship).^45^ These relationships are based on preclinical and small-numbered clinical studies with methodological limitations. Ultimately, we agree with Seibel *et al.*^28^ that TDM has a role in preventing nephrotoxicity (rather than ensuring efficacy). All the included PK studies looking at *C*_max_ at doses of 2.5–10 mg/kg/day appear to be above target levels of 25–50 mg/L. However, patients under 20 kg may require higher doses of up to 12 mg/kg/day.^25,27,28^ The measure of the efficacy of IFD antifungal therapy may be challenging due to discordant clinical, radiological or mycological data and a variable period of patient evaluation. Children treated for IFD are often immunocompromised, and the response to treatment may rely on host responses, the capacity of immune reconstitution after chemotherapy, and comorbidities.^46^ L-amb-induced nephrotoxicity may be easier to measure and control for confounders with novel biomarkers.^47,48^

The concluding answer to this research question is, no, we are not there yet with L-amb TDM in children. Of the six included studies, only two discussed the utility of TDM but did not investigate it. Hong *et al.*^25^ concluded that more work is needed to clarify the impact of TDM. Seibel *et al.*^28^ concluded that TDM is likely to be valuable in preventing toxicity. The values may be pronounced in patients on high doses of L-amb, long durations (>14 days) and concurrent nephrotoxins. Despite the accumulating evidence of PK variability potentially warranting L-amb TDM, our systematic review has not identified the evidence to support routine TDM using *C*_max_/MIC = 25–50 for optimal efficacy and AUC_24_ < 600 mg·h/L to minimize nephrotoxicity. We need adequately powered clinical studies with better definitions of treatment outcomes and AKI, consistent use of MIC breakpoint values as a denominator, and improved study designs that include the aspects contributing to L-amb non-linear PK.

## Supplementary Material

dkae003_Supplementary_Data

## Supplementary data

Tables S1 to S3 are available as Supplementary data at *JAC* Online.
