# Supplementary material for: Therapeutic drug monitoring of liposomal amphotericin B in children. Are we there yet? A systematic review
Source: J Antimicrob Chemother. 2024 Jan 22;79(4):703–11. doi: 10.1093/jac/dkae003 (PMC10984953; doi:10.1093/jac/dkae003)
Supplement: dkae003_Supplementary_Data [file dkae003_supplementary_data.docx]

**Supplement**

**Table S1. EMBASE^a^ systematic review search strategy**

| Set | Search statement |
| --- | --- |
| 1 | ((“Amphotericin B)” AND “(Drug monitoring” OR “Pharmacokinetics” OR “Pharmacodynamics” OR “drug concentration”) AND (“child” OR “adolescent”)) ^49^ |
| 2 | limit 1 to yr="1984 -Current" |
| 3 | limit 2 to human |
| 4 | limit 3 to (infant <to one year> or child <unspecified age> or preschool child <1 to 6 years> or school child <7 to 12 years> or adolescent <13 to 17 years>) |

^a^ Search of EMBASE conducted via OVID on 21st June 2023

**Table S2.** PUBMED^a^ systematic review search strategy

| Set | Search statement |
| --- | --- |
| 1 | ("amphotericin b"[MeSH Terms] OR "amphotericin b"[All Fields]) OR ("amphotericin b" [MeSH Terms] OR "amphotericin b"[All Fields]) AND ("pharmacokinetics"[Subheading] OR "pharmacokinetics"[All Fields] OR "pharmacokinetics"[MeSH Terms]) |
| Filter | Humans, English, Child: birth-18 years |

^a^ Search of PUBMED conducted on 21st June 2023

**Table S3:** **Critical Appraisal of Clinical Pharmacokinetics (CACPK) studies quality assessment tool.**

Derived from Soliman *et al.* 2022 ^21^:

| Critical Appraisal of Clinical Pharmacokinetics (CACPK) assessment items | |
| --- | --- |
| 1. Appraising Background |  |
| 1. Was a clear description of the objectives of the study provided?  • Authors should provide a clear statement of the objectives of the research to clarify the purpose and the scope of the study. | Yes  No  I Do Not Know  Not Applicable  Comments: ____________ |
| 2. Was a clear and comprehensive rationale provided to support the purpose of the study? | Yes  No  I Do Not Know  Not Applicable  Comments: ____________ |
| Appraising Study Design and Experimental Methods |  |
| 3. Was the chosen study design appropriately selected and justified? | Yes  No  I Do Not Know  Not Applicable  Comments: ____________ |
| 4. Was the dosing (i.e. dose, route of administration, and dosing interval) of the drug in the study justified for the intended study?  Examples: • Authors should justify the use of single-dose versus steady-state analysis. | Yes  No  I Do Not Know  Not Applicable  Comments: ____________ |
| 5. Were the outcome measures endpoints of the study appropriate to address the objectives of the study? | Yes  No  I Do Not Know  Not Applicable  Comments: ____________ |
| 6. Were the exclusion criteria of participants included AND appropriate for the intended outcomes of the study?  • The exclusion criteria should be relevant to assist with decreasing significant confounders (e.g. co-administration of drugs, organ impairment, and special populations) that may impact outcomes. | Yes  No  I Do Not Know  Not Applicable  Comments: ____________ |
| 7. Where applicable, were the relevant baseline characteristics of the participants adequately described? Examples:  • Sex, race, age, weight, height, concomitant disease, administered medications, smoking status, pregnancy, severity of illness that may affect pharmacokinetic parameters, renal function, and hepatic function. | Yes  No  I Do Not Know  Not Applicable  Comments: ____________ |
| 8. Were plausible interacting covariates described a priori or in post hoc evaluation?  Examples:  • Demographic variables, laboratory values, concomitant medications, and relevant disease states to the drug being studied. | Yes  No  I Do Not Know  Not Applicable  Comments: ____________ |
| 9. Was the description of the used biological sample analytical methods sample analysis methods or citations of prior validation studies provided in the publication or affiliated appendix?  Examples:  • Chromatography type.  • Detection type. • Assay characteristics: mobile phase composition, gradient and flow rate, chromatographic column (packing material, dimensions). • Analytical runtime. • Operating temperature.  • Detection parameters. • Validation method: specificity, recovery, linearity and sensitivity, the stability of the assay and its reproducibility. Refer also to EMA/FDA guidelines for bioanalytical method validation. | Yes  No  I Do Not Know  Not Applicable  Comments: ____________ |
| 10. Was the method of data sampling of analytics appropriate for the study? Examples:  • First versus. second order absorption, and lag time.  • Evaluating for nonlinearity requires multiple dose levels and a complete profile is recommended.  • Researchers obtain these data from previously conducted studies with completed concentration-time profile (e.g. phase I studies).  • The method of data sampling should reference previously validated quantitative bioanalytical methods and if those are not available then the full description or defense of data sampling should be included. | Yes  No  I Do Not Know  Not Applicable  Comments: ____________ |
| 11. Was a clear description of the sampling site provided and justified?  Examples:  • Sampling site should be consistent for all subjects in the study. | Yes  No  I Do Not Know  Not Applicable  Comments: ____________ |
| 12. Was the number of half-lives elapsed within the sampling period appropriate for the analysed drug?  Examples:  • Sampling interval should not exceed the expected half-life of the studied exponential phase (fast distribution, slow distribution and elimination). | Yes  No  I Do Not Know  Not Applicable  Comments: ____________ |
| 13. Were sample storage conditions appropriate and described in a manner that could be accurately replicated?  Examples:  • Sample storage, temperature, use and description of anticoagulants, stabilisers, centrifugation etc. | Yes  No  I Do Not Know  Not Applicable  Comments: ____________ |
| 14. If applicable, was there a clear description of the pharmacokinetic model, its development, validation and justification for use?  It is recommended to provide the following details about the selected modelling process: • Description of studies from which dataset was driven • Model structure • Validated software for the pharmacokinetic analysis • Criteria for accepting valid model’s parameters  • Fitting procedure defined prior to the initiation of the analysis. • A reasonable assumption based on which the scheme for weighting is considered to be appropriate and the transformation of data [e.g. logarithmic transformation to achieve the homoscedastic (constant) variance requirements] should be provided. | Yes  No  I Do Not Know  Not Applicable  Comments: ____________ |
| 15. Was the described population pharmacokinetic approach validation method appropriate for the analysis? 1- Basic internal method 2- Advanced internal method 3- External model evaluation | Yes  No  I Do Not Know  Not Applicable  Comments: ____________ |
| 16. Were the essential pharmacokinetic parameters required to make the results applicable in clinical settings included?  Examples: • Total clearance (CL), Hepatic clearance, Renal clearance, Volume of distribution at steady state (Versuss), Blood/plasma concentration ratio, Terminal half-life (t1/2 Z), Fraction of the unbound drug in plasma (fu), Absorption rate constant (Ka),Cmin, Cmax, tmax,, AUC, etc. | Yes  No  I Do Not Know  Not Applicable  Comments: ____________ |
| 17. Were the pharmacokinetic equations used to calculate the patient’s pharmacokinetic parameters presented or cited within the article?  Examples:  • Equations used to calculate the following pharmacokinetic parameters: creatinine clearance, body weight calculations, Michaelis Menten, Volume of distribution | Yes  No  I Do Not Know  Not Applicable  Comments: ____________ |
| Appraising Applied Statistics |  |
| 18. Were the chosen statistical tests and software to perform the statistical analysis appropriate to achieve the study objectives? | Yes  No  I Do Not Know  Not Applicable  Comments: ____________ |
| 19. Were all patients enrolled in the study accounted for? Examples:  • Description of patient screening, enrolment, run-in or wash out phases, study period and follow-up periods are adequately described. Any loss to follow-up or withdrawals are described. | Yes  No  I Do Not Know  Not Applicable  Comments: ____________ |
| 20. In the event of missing data or outliers, was the process for analysis justified and appropriate? | Yes  No  I Do Not Know  Not Applicable  Comments: ____________ |
| 21. Were appropriate summary statistics to describe centrality and variance used to present the pharmacokinetic results? Examples:  • Descriptive statistics such as confidence interval, standard deviation, mean, median, range, interquartile range, standard error and trimmed range | Yes  No  I Do Not Know  Not Applicable  Comments: ____________ |
